# Chronic spontaneous urticaria preceded by localized insulin reactions: case report

**DOI:** 10.1177/2050313X241248383

**Published:** 2024-04-22

**Authors:** Zoha K. Momin, Jeffrey M. Chambliss

**Affiliations:** Division of Allergy and Immunology, Department of Pediatrics, University of Texas Southwestern Medical Center, Dallas, TX, USA

**Keywords:** Type 1 diabetes, chronic spontaneous urticaria, insulin, urticaria

## Abstract

Chronic spontaneous urticaria presents with wheals and/or angioedema for >6 weeks without any specific triggers. The incidence of chronic spontaneous urticaria is increased in patients with comorbid autoimmune conditions. Here, we present a case of chronic spontaneous urticaria in a 9-year-old with type 1 diabetes and autoimmune thyroid disease who first presented with insulin pump site reactions concerning an insulin-related allergy. The patient was successfully treated with antihistamines and later immunosuppression with resumption of insulin pump therapy and remission of chronic spontaneous urticaria symptoms 18 months after onset.

## Introduction

Chronic spontaneous urticaria (CSU), previously known as chronic idiopathic urticaria, is characterized by the development of wheals and/or angioedema for >6 weeks in the absence of any identifiable triggers.^1^ While its underlying pathogenesis is not yet fully understood, in a significant subset of patients with CSU, autoantibodies can be detected and may contribute to symptoms.^2,3^ Many patients with CSU often have other autoimmune conditions, including autoimmune thyroid disease and type 1 diabetes (T1DM).^4–6^

Overlapping dermatologic and systemic presentations between CSU and other autoimmune and allergic conditions can make diagnosis challenging. In diabetic patients receiving long-term insulin therapy, CSU may be difficult to distinguish from insulin allergy, especially in the acute setting.^7–9^ Careful history taking, focused particularly on the timing of symptoms, may distinguish a mechanism of reaction and guide evaluation, as insulin itself, preservatives, and pump equipment can all be implicated in reactions.^10^ Here, we present a case of CSU initially concerning insulin allergy in a pediatric patient with T1DM and autoimmune thyroid disease.

## Case report

A 9-year-old Caucasian female diagnosed with T1DM and Grave’s disease 3 years prior presented to the allergy/immunology clinic in 2019 due to concern for insulin reactions. Her Grave’s disease was noted to be in remission and off therapy at presentation. At the time of T1DM diagnosis, she was initiated on lispro and glargine insulin therapy and transitioned to a continuous insulin pump 1 year after diagnosis without issue. However, over the preceding 5 months, she started to develop a firm, pruritic raised erythematous nodule at the pump site within 12 h of use, reproducible at different pump locations. Despite insulin pump removal, erythema surrounding the pump site persisted for 3 days post-removal. In the week prior to evaluation, she switched to insulin aspart and glargine subcutaneously, which did not improve local symptoms. At her initial visit, a firm nodule with surrounding ecchymosis was appreciated from the most recent pump site. Injection sites from the day prior were raised and erythematous, but injection sites from 2 h prior were unremarkable. The patient and family did not report any history of shortness of breath, nausea, vomiting, diarrhea, dizziness, or syncope associated with insulin administration. Further, the patient had no history of other atopic conditions or recurrent rash. Laboratory evaluation was unremarkable for other underlying causes (see Table 1).

**Table 1.** Diagnostic workup at the time of initial clinical presentation. Complete blood cell count with differential (not listed here) was within normal limits.

Lab	Normal range	Patient result
Hgb A1C	<5.7%	9.4%*
Free T4	0.89–1.76 ng/dL	2.13*
T3	28–294 ng/dL	277
Thyroid stimulating hormone	0.4–5.56 microIU/mL	<0.008*
Luteinizing hormone	0–0.70 milli-IU/mL	<0.07
Follicle-stimulating hormone	0.4–4.4 milli-IU/mL	3.4
Estradiol	<15 pg/mL	6.3
Insulin autoantibody	0.0–0.4 U/mL	>50.0*
Thyroglobulin antibody	0.0–1.0 IU/mL	>2374.0*
Thyroid peroxidase antibody	<35.0 IU/mL	>1542.0*
Thyroid stimulating immunoglobulin	<122%	112
C3 complement	80–150 mg/dL	133
C4 complement	13–38 mg/dL	23.8
IgA	41–297 mg/dL	175.3
Tissue transglutaminase IgA	0–9.9 EliA/dL	0.2
C-reactive protein	0–1 mg/dL	<0.4
Erythrocyte sedimentation rate	0–20 mm/hr	6

* Denotes lab values out of normal range.

Given the delayed onset of symptoms, IgE-mediated insulin allergy seemed unlikely, and symptomatic management was optimized. The patient was started on cetirizine 10 mg twice daily and topical triamcinolone 0.1% applied post-injection without improvement. At follow-up, the topical steroid potency was increased, and topical cromolyn, as well as other insulin formulations not previously utilized, were trialed without symptom resolution.

Due to the persistence of these symptoms, lesion punch biopsy and subcutaneous metacresol skin testing were conducted to evaluate for vasculitis and insulin preservative reactions, respectively. Punch biopsy demonstrated superficial and deep perivascular and interstitial infiltrate of eosinophils, lymphocytes, and neutrophils, compatible with an urticarial reaction. Metacresol skin testing was negative. She also began to intermittently experience generalized, pruritic maculopapular eruptions with occasional mild facial angioedema without other systemic symptoms, initially concerning for serum sickness-like reaction. Given negative workup and progressive symptoms, she was admitted to the local pediatric hospital for attempted IV insulin to bypass subcutaneous injections for a brief period while providing immunosuppression with mycophenolate and systemic steroids. While inpatient, she developed a generalized, erythematous non-pruritic rash following IV insulin that responded well to diphenhydramine. At discharge, she was transitioned back to an insulin pump with a 10-day prednisone taper, mycophenolate 300 mg twice daily, and cetirizine 10 mg twice daily. Localized pump site reactions were mildly improved with this regimen; however, over the ensuing months, she developed recurrent widespread urticarial plaques 2–4 times weekly. H2 antihistamines were added to her treatment regimen, and mycophenolate was increased to 500 mg AM and 250 mg PM, dosing with hydroxyzine as needed.

The patient was continued on this regimen and experienced significantly fewer episodes of urticaria. Given clinical improvement, the patient was tapered off mycophenolate 10 months after initiation. On her modified treatment regimen, she experienced urticaria and angioedema 1–2 times monthly, namely on antihistamine-free days. Her pump site reactions were managed with topical pre-pump placement of 0.05% clobetasol. Eighteen months after initial presentation, the patient reported few to no insulin pump site reactions and mild maculopapular eruptions only on days she did not take antihistamines. Antihistamines were slowly tapered over the course of a year. Thirty months after initiation of treatment, the patient had stopped all antihistamines with only intermittent cetirizine usage in rare cases of urticaria. Following remission of her urticaria, she had one mild pump site reaction and was doing well with an insulin pump.

## Discussion

Chronic urticaria can be divided into inducible and spontaneous subtypes that can be clinically distinguished based on identification of specific triggers. The pathogenesis of CSU is driven by mast cell activation and subsequent release of histamine and other preformed mediators, with IL-31 and IL-33 also involved and downstream activation of effector cells including basophils, eosinophils, and T lymphocytes, including Th2 and Th17 cells.^1,11,12^ The resulting inflammatory response increases vascular permeability and manifests as urticarial wheals and/or angioedema.^2,3^ While immune signaling dysregulation may underlie CSU development, some studies approximate >50% of CSU patients have autoimmune mechanisms involved.^2,3^

Regardless of etiology, CSU has been associated with autoimmune comorbidities, including increased odds of autoimmune hypothyroidism, hyperthyroidism, and associated anti-thyroid antibodies in patients with chronic urticaria.^4^ In an age-matched study with non-T1DM patients, children <18 years of age with T1DM had a 3.62-fold higher risk of acute or chronic urticaria development.^5^ Due to autoimmune islet cell destruction, patients with T1DM require lifelong insulin replacement therapy, making insulin reaction evaluation critical. Insulin reactions can present as immediate, type I IgE-mediated hypersensitivity or delayed, localized type IV reactions. Immediate reactions are often diagnosed by skin prick testing or testing for specific IgE antibodies against various insulin types, although these can also be incidentally present in insulin-treated individuals. Delayed reactions are more often associated with additives, including zinc, metacresol, and protamine in insulin preparations or infusion pump equipment, including tubing and adhesives.^10^ Type III hypersensitivity reactions, such as serum-sickness-type reactions to insulin, have also been described.^7–9^ The wide range of insulin associated reactions and increased incidence of CSU in T1DM patients can make it challenging to distinguish the two diagnoses.

In the case above, despite initial presentation with localized injection site reactions, development of more generalized urticaria and angioedema in the absence of other systemic symptoms was consistent with a diagnosis of CSU. Initially, multiple different insulin preparations were tried with continued localized symptoms, with and without use of a pump. Desensitization with IV insulin may have a role in IgE-mediated reactions but was pursued in this patient with a goal of cutaneous rest, albeit without significant clinical improvement. Given negative metacresol testing and continued reactions without a pump utilized, a type IV hypersensitivity reaction to preparation preservatives or pump equipment seemed unlikely and patch testing was not pursued. Additionally, skin biopsy suggested an urticarial reaction, and the patient responded to aggressive antihistamine and immunosuppressant therapy. Omalizumab has been used successfully in managing CSU, including in a patient with concurrent T1DM and is approved for patients 12 and older for CSU but has been used for this indication off-label in younger children.^1,13^

CSU eruptions present with pruritic wheals and/or subcutaneous or submucosal angioedema, typically lasting 24 h or less at individual locations. Eruptions can recur daily or intermittently, but the condition can persist for years on end.^3^ Bruising or lesions lasting greater than 24 h should prompt consideration of vasculitis and periodic fever or other systemic symptoms consideration of autoinflammatory diseases.^14^ Still, CSU is often self-limiting with spontaneous resolution of symptoms. In a pediatric chronic urticaria population, a 10% yearly rate of complete symptom resolution has been reported.^15^ Treatment of CSU proves challenging as therapeutic response varies across individuals. Long-acting, second-generation antihistamines are the first-line agents for CSU management. Increasing up to four times the standard dosing is recommended in a stepwise manner. If symptom control remains suboptimal, the biological agent omalizumab may be added or considered immunosuppressant, such as cyclosporine.^1,16^ In this case, mycophenolate was chosen after shared decision making with the family regarding potential side effects. While Vitamin D and the microbiome are involved in immune responses,^17^ further studies are needed to better understand their contributions to the management of CSU.

## Limitations

This study is limited by its retrospective nature and case report design focusing on a single patient’s presentation.

## Conclusion

Our case highlights the challenges and considerations in evaluating a patient with insulin reactions as well as management strategies for CSU. Thorough clinical history and diagnostic workup can help distinguish CSU from suspected insulin allergy and allow safe continuation of insulin therapy in diabetic patients.
